# Constructing a seventeen-gene signature model for non-obstructive azoospermia based on integrated transcriptome analyses and WGCNA

**DOI:** 10.1186/s12958-023-01079-5

**Published:** 2023-03-21

**Authors:** Yinwei Chen, Penghui Yuan, Longjie Gu, Jian Bai, Song Ouyang, Taotao Sun, Kang Liu, Zhao Wang, Chang Liu

**Affiliations:** 1 Reproductive Medicine Center, Tongji Hospital, Tongji Medical College, Huazhong University of Science and Technology, Wuhan, 430030 Hubei China; 2 Department of Urology, the First Affiliated Hospital of Zhengzhou University, Zhengzhou, 450000 Henan China; 3 Department of Urology, Tongji Hospital, Tongji Medical College, Huazhong University of Science and Technology, Wuhan, 430030 Hubei China; 4 Department of Urology, First Affiliated Hospital, School of Medicine, Shihezi University, Shihezi, 832008 Xinjiang China; 5 Department of Urology, Xiangya Hospital, Central South University, Changsha, 410000 Hunan China; 6 Reproductive Medicine Center, Nanjing Drum Tower Hospital, The Affiliated Hospital of Nanjing University Medical School, Nanjing, 210008 Jiangsu China

**Keywords:** Non-obstructive azoospermia, Sperm, Integrated analysis, Weighted correlation network analysis, LASSO regression

## Abstract

**Background:**

Non-obstructive azoospermia (NOA) affects approximately 1% of the male population worldwide. The underlying mechanism and gene transcription remain unclear. This study aims to explore the potential pathogenesis for the detection and management of NOA.

**Methods:**

Based on four microarray datasets from the Gene Expression Omnibus database, integrated analysis and weighted correlation network analysis (WGCNA) were used to obtain the intersected common differentially expressed genes (DESs). Differential signaling pathways were identified via GO and GSVA-KEGG analyses. We constructed a seventeen-gene signature model using least absolute shrinkage and selection operation (LASSO) regression, and validated its efficacy in another two GEO datasets. Three patients with NOA and three patients with obstructive azoospermia were recruited. The mRNA levels of seven key genes were measured in testicular samples, and the gene expression profile was evaluated in the Human Protein Atlas (HPA) database.

**Results:**

In total, 388 upregulated and 795 downregulated common DEGs were identified between the NOA and control groups. ATPase activity, tubulin binding, microtubule binding, and metabolism- and immune-associated signaling pathways were significantly enriched. A seventeen-gene signature predictive model was constructed, and receiver operating characteristic (ROC) analysis showed that the area under the curve (AUC) values were 1.000 (training group), 0.901 (testing group), and 0.940 (validation set). The AUCs of seven key genes (REC8, CPS1, DHX57, RRS1, GSTA4, SI, and COX7B) were all > 0.8 in both the testing group and the validation set. The qRT-PCR results showed that consistent with the sequencing data, the mRNA levels of RRS1, GSTA4, and COX7B were upregulated, while CPS1, DHX57, and SI were downregulated in NOA. Four genes (CPS1, DHX57, RRS1, and SI) showed significant differences. Expression data from the HPA database showed the localization characteristics and trajectories of seven key genes in spermatogenic cells, Sertoli cells, and Leydig cells.

**Conclusions:**

Our findings suggest a novel seventeen-gene signature model with a favorable predictive power, and identify seven key genes with potential as NOA-associated marker genes. Our study provides a new perspective for exploring the underlying pathological mechanism in male infertility.

**Supplementary Information:**

The online version contains supplementary material available at 10.1186/s12958-023-01079-5.

## Introduction

Infertility is defined as the failure to achieve a pregnancy after one year of regular unprotected sexual intercourse [1]. Approximately 10 to 15% of couples deal with infertility issues globally, and nearly 50% of these cases are caused by male factors [2, 3]. Moreover, current evidence points out that male infertility has shown a steadily progressive increase in recent decades [4]. Azoospermia, a disease in which not a single sperm is found in three consecutive ejaculations, is a major cause of male infertility. According to the condition of active spermatogenesis in the testis, azoospermia can be classified into two categories obstructive azoospermia (OA) and non-obstructive azoospermia (NOA). NOA is a disease characterized by testicular failure due to the unknown genetic, developmental, hormonal, environmental, and other reasons [5–7].

NOA, a disorder in which spermatozoa is absent in the ejaculate, is the most severe type of male infertility [8, 9]. In the past, most NOA patients have received donor sperm treatment to achieve pregnancy via assisted reproductive techniques [10]. In 1994, a novel approach, namely, testicular sperm extraction (TESE), which retrieved sperm from the testes of NOA patients, made it possible for these patients to have their own offspring [8]. Since then, the combination of testicular sperm extraction and intracytoplasmic sperm injection (ICSI) has become the first-line treatment for men with NOA [11]. Advances in techniques (e.g. micro-TESE) allow sperm-containing regions to be identified before their removal, resulting in a higher successful sperm retrieval rate. However, the successful retrieval of spermatozoa in NOA occurs in only approximately 50% of cases [12]. This is due to the heterogeneous nature of NOA, which affects spermatogenesis. The subtype of NOA includes hypospermatogenesis, maturation arrest, and Sertoli cell-only syndrome [7]. Therefore, investigating the pathogenesis and molecular signature has great clinical significance for the management of NOA.

Given the rapid progression of microarray and high-throughput sequencing, characterizing the genetic changes during spermatogenesis is of increasing interest to researchers. For instance, Cioppi et al. reported 38 candidate genes involved in NOA based on impaired reproductive phenotypes in humans and mice [7]. In addition, the European Association of Urology (EAU) guidelines recognize the significance of screening karyotype abnormalities and Y chromosome microdeletions for patients with NOA [13, 14]. Using genome-wide gene expression analysis, single nucleotide polymorphism (SNP) of *ART3* was identified to be significantly enriched in NOA patients [15]. Although growing evidence has confirmed that multiple genetic and mutational defects are involved in NOA, there are still many mysteries about the pathogenesis of spermatogenesis failure. For example, genome-wide association studies (GWAS) have been used as a promising tool to identify genetic variation in NOA. A GWAS on a European NOA cohort demonstrated significant NOA-associated SNPs in the *KDM3A* and *TAS2R38* genes [16]. Nevertheless, a well-powered GWAS containing 2927 NOA patients and 5734 control participants in East Asians, revealed that significant NOA-associated genomic regions were located in the genes *PRMT6*, *PEX10*, and *SOX5* [17]. Another GWAS of Asian patients found that the significant NOA SNPs resided in the genes *CHD2*, *GNAO1*, and *BCL2* [18]. Thus, the pathogenesis is highly complicated and cannot be explained by the abnormalities caused by a single gene. It is expected that utilizing more systematic biomarkers with high statistical efficiency may show greater predictive value and clinical significance.

Least absolute shrinkage and selection operation (LASSO) is a widely used regression-based methodology in the current statistical field. It provides a greater prediction accuracy and increases model interpretation over other regression models, and it has a large number of covariates and a unique feature penalizing coefficient [19–21]. This method is conducive to more comprehensive screening and integration of molecular differences, and helps us better comprehend the causes underlying spermatogenic diseases.

In the present study, a total of four microarray datasets in the GEO database were integrated and systematically analyzed to determine the NOA-associated hub genes and signal functions via multilayer screening of bioinformatics methods. In addition, a seventeen-gene signature model was constructed to predict NOA. Our study provides insight into the exploration of underlying pathogenesis and the identification of potential therapeutic targets for NOA and male infertility.

## Materials and methods

### Data acquisition

The gene expression profile datasets regarding NOA were obtained from the Gene Expression Omnibus (GEO) database (http://www.ncbi.nlm.nih.gov/geo/) (GSE4797, GSE6023, GSE45885, GSE45887, GSE9210, and GSE145467) on December 15^th^, 2021. The six datasets were updated from 2012 to 2020, and come from different European and Asian countries (Supplementary Table 1). After screening, 173 individuals, including 42 samples with normal spermatogenesis (the control group) and 131 samples with NOA (the NOA group), were utilized for further analysis. To create an accurate and reliable model for NOA prediction, datasets were divided into a training group (GSE4797, GSE6023, GSE45885, and GSE45887), testing group (GSE9210), and validation set (GSE145467).

### Integrated analysis of DEGs

We used R software (Version 3.6.3) to process the data. Primary GEO matrix files were downloaded and normalized by the R package “limma” [22]. Limma is an R package for processing data, especially for gene expression experiments. The raw matrixes were regularly normalized and evaluated to see if they needed log2 transformation (Supplementary Fig. 1). The subsequent steps included the conversion of the probe name and gene name, averaging genes with the same name, and removing defect values. Strawberry-perl software (Version 5.30) was utilized to merge the four matrixes (the GSE4797, GSE6023, GSE45885, and GSE45887 datasets) continuously. Then, the merged training matrix was used by the R package “sva” [23] to remove the batch effect between arrays. Using the integrated dataset, we calculated the differentially expressed genes (DEGs) between the NOA and control groups via the “limma” package. We used the functions “contrasts.fit” and “eBayes” to calculate the |log2 fold-change| (|log2FC|) and adjusted *P* value of each gene. The selected criteria for DEGs included |log2FC|> 0.5 and adjusted *P* value < 0.05. DEGs with log2FC > 0.5 were regarded as upregulated DEGs, while DEGs with log2FC < -0.5 were regarded as downregulated DEGs.

### Weighted correlation network analysis (WGCNA)

The coexpression network for all genes was constructed via weighted correlation network analysis (using the R package “WGCNA” [24]). The clinical information extracted from the training group was the same as “NOA” and “control”. The value of the soft-power threshold (power = 6) was used to create the weighted adjacency matrix and gain the optimal scale-free topology model. Then, the topological overlap measure (TOM) was transformed from the weighted adjacency matrix. Hierarchical clustering was performed to construct a clustering dendrogram. Each gene module size contained at least 50 genes. The threshold to merge similar modules was set to 0.3, and *P* value < 0.05 was considered significant.

### Venn intersection

To obtain genes more enriched in NOA, we combined the GEO_DEGs and WGCNA_genes. Later, we took the intersection to obtain common genes between them using the R package “ggvenn” (https://cran.r-project.org/web/packages/ggvenn/index.html).

### Functional enrichment analysis for common DEGs

Gene Ontology (GO) analysis is a major informatic method to find significantly enriched biological functions. The analysis includes GO terms for biological process, cellular component, and molecular function. The R package “clusterProfiler” [25] was utilized to perform these analyses for common genes, and the R package “ggplot2” [26] was utilized to visualize GO clusters. We set adjusted *P* < 0.05 and *q* < 0.05 as statistical significance.

### Construction of protein–protein interaction network

Significant common DEGs were put into the STRING database (https://string-db.org/) to identify the protein–protein interaction (PPI) network. We removed the disconnected nodes and built the PPI network using Cytoscape 3.8.0 software (https://cytoscape.org/). The cytoHubba plug-in was used to select key modules involved in NOA. The MCODE score was set to > 5.0.

### Gene set variation analysis

To detect pathway activity changes regarding NOA, we performed gene set variation analysis (GSVA) using the R packages “GSVA” [27] and “GSEABase” [27]. By utilizing the Kyoto Encyclopedia of Genes and Genomes (KEGG) hallmark gene sets as the reference gene set, we identified the commonly activated/inhibited pathways involving NOA. The selection criteria included adjusted *P* < 0.05 and |log2FC|> 0.2. The volcano plot and heatmap were made using the R packages “limma” and “ggplot2”.

### Lasso model construction

The common DEGs were further screened to construct the predictive model and identify potential prediction-associated genes. The LASSO model is a regression method that performs good variable selection and regularization, and we applied LASSO regression analysis to the training group using the R packages “glmnet” [28] and “limma”. Next, we developed a seventeen-gene signature-based predictive model. The risk score of each sample was calculated as follows: Risk score = ∑Exp_i_ (Expression_i_) × β_i_. Exp indicates the expression value of a single gene, and β indicates the regression coefficient of a single gene.

### Receiver operating characteristic curve (ROC) evaluation

To evaluate the performance of the LASSO model, we used the risk score to predict the NOA phenotype of each sample using the R package “timeROC” (https://cran.r-project.org/web/packages/timeROC/). In addition, we used samples from the integrated training group, testing group (GSE9210), and validation set (GSE145467) to further validate the predictive value of this model. The seventeen potential predictive genes were also screened according to their area under the ROC curves (AUCs). We selected seven key genes with AUCs > 0.8 in both the testing and validation groups and visualized their curves by the R package “ggplot2”.

### Verification and localization of predictive genes

We further verified the expression profile of seven key genes in the integrated training dataset. We measured the expression difference between the NOA and control groups using the R packages “limma” and “ggpubr” (https://cran.r-project.org/web/packages/ggpubr/). *P* < 0.05 or *P.adjust* < 0.05 were regarded as statistically significant. Next, the immunohistochemical data and single-cell transcriptome data were downloaded from the Human Protein Atlas (HPA) database (https://www.proteinatlas.org/) to determine the localization characteristics of key genes in human testis. The concrete data source from the HPA database was shown in Supplementary Tables 2 and 3.

### Participants

Six couples with male infertility attending Tongji Hospital were recruited in the study. Of these couples, three male patients with NOA were divided into the NOA group, while another three male patients with OA were divided into the control group. For NOA patients, the inclusion criteria included the following: 1) at least three semen analyses after centrifugation that revealed no sperm; 2) no obstruction/developmental malformation-associated evidence in the epididymis, vas deferens and ejaculatory duct; and 3) impaired spermatogenic function after examination of testicular tissues by testicular biopsy. For OA patients, the inclusion criteria included the following: 1) the same as NOA Item 1); 2) obstruction/developmental malformation-associated evidence in the epididymis, vas deferens and ejaculatory duct; and 3) normal spermatogenic function by testicular biopsy. Written consent from each individual was obtained. All procedures were approved by the Institutional Research Ethics Committee of Huazhong University of Science and Technology REC No. (2017(04)). Our study abided by the Declaration of Helsinki.

### Specimen collection and storage

Testicular biopsy was performed for these six male patients. In brief, the surgical area was cleaned, and local anesthesia was performed. A small portion of the testicular tissue was taken using a special needle. The tissue was immediately preserved in RNALater™ RNA Stabilization Reagent for Animal Tissue (R0118FT, Beyotime, Haimen, China), and then frozen at -80 °C.

### Quantitative real-time polymerase chain reaction (qRT-PCR) analysis

To examine the expression of genes obtained from the sequencing results, we determined their mRNA levels in NOA and normal spermatogenesis samples. According to the instructions, total RNA from six testicular tissues was extracted using RNA-easyTM Isolation Reagent (R701-02, Vazyme, Nanjing, China), and the RNA samples were synthesized into cDNA using a Hifair® II 1st Strand cDNA Synthesis Kit (11121ES60, Yeasen, Shanghai, China). We performed qRT-PCR and analyzed gene expression as previously described [29]. The *GAPDH* gene was used as the endogenous reference. Detailed information regarding primer sequences was shown in Supplementary Table 4.

### Statistical analysis

All quantitative variables are presented as the mean ± standard deviation (SD), and Student’s t test was performed to evaluate statistical significance using SPSS version 23.0 (IBM Corporation, Armonk, NY, USA). A *P* value < 0.05 was considered statistically significant.

## Results

### Identification of DEGs in NOA

**Fig. 1:**
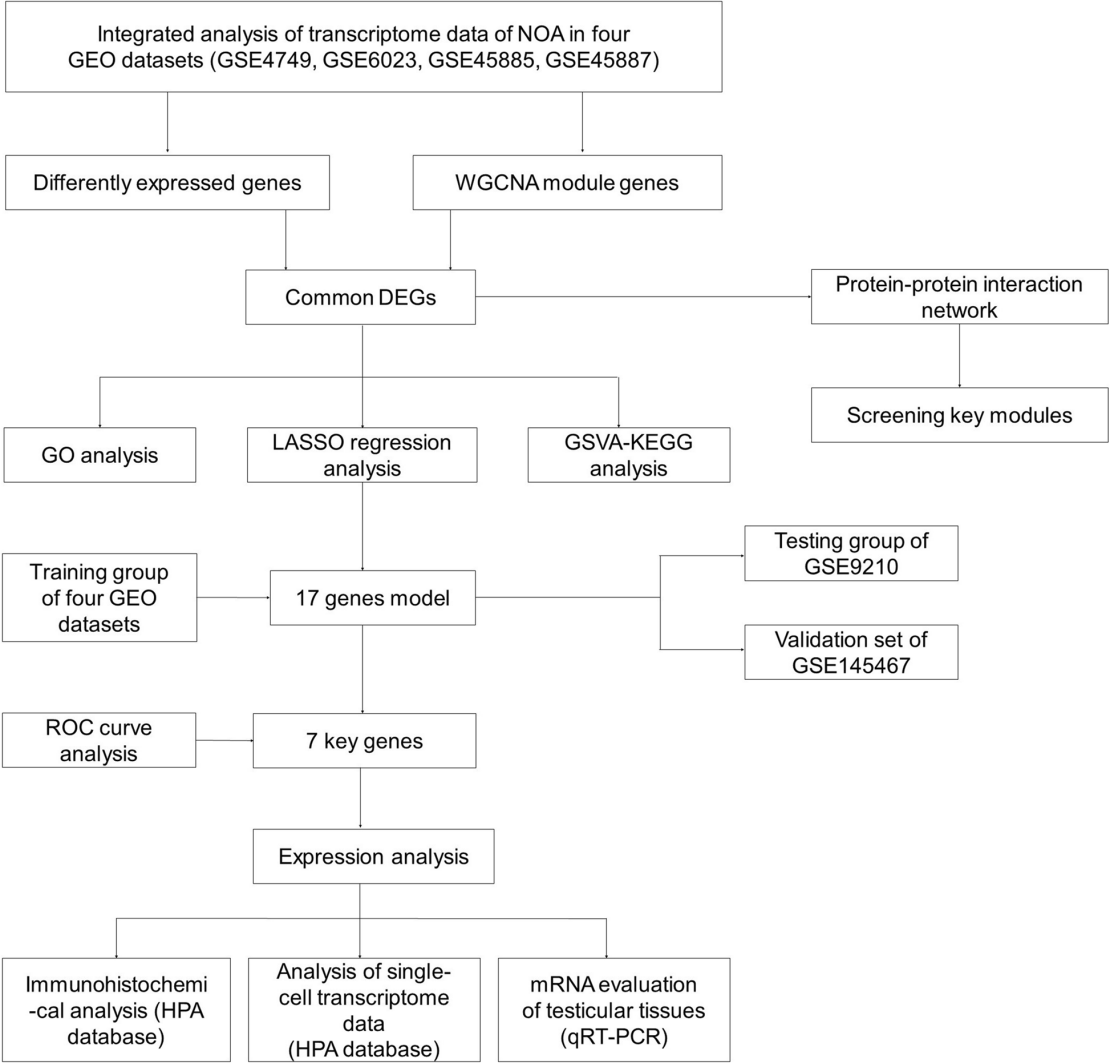
The flow diagram of study strategy. NOA: non-obstructive azoospermia; GEO: Gene Expression Omnibus; GSE: GEO Series; WGCNA: weighted gene coexpression network analysis; GO: Gene Ontology; LASSO: Least Absolute Shrink and Selection Operator; KEGG: Kyoto Encyclopedia of Genes and Genomes; GSVA: Gene Set Variation Analysis; ROC: Receiver Operating Characteristic; HPA: Human Protein Atlas; qRT-PCR: quantitative real-time polymerase chain reaction

**Fig. 2:**
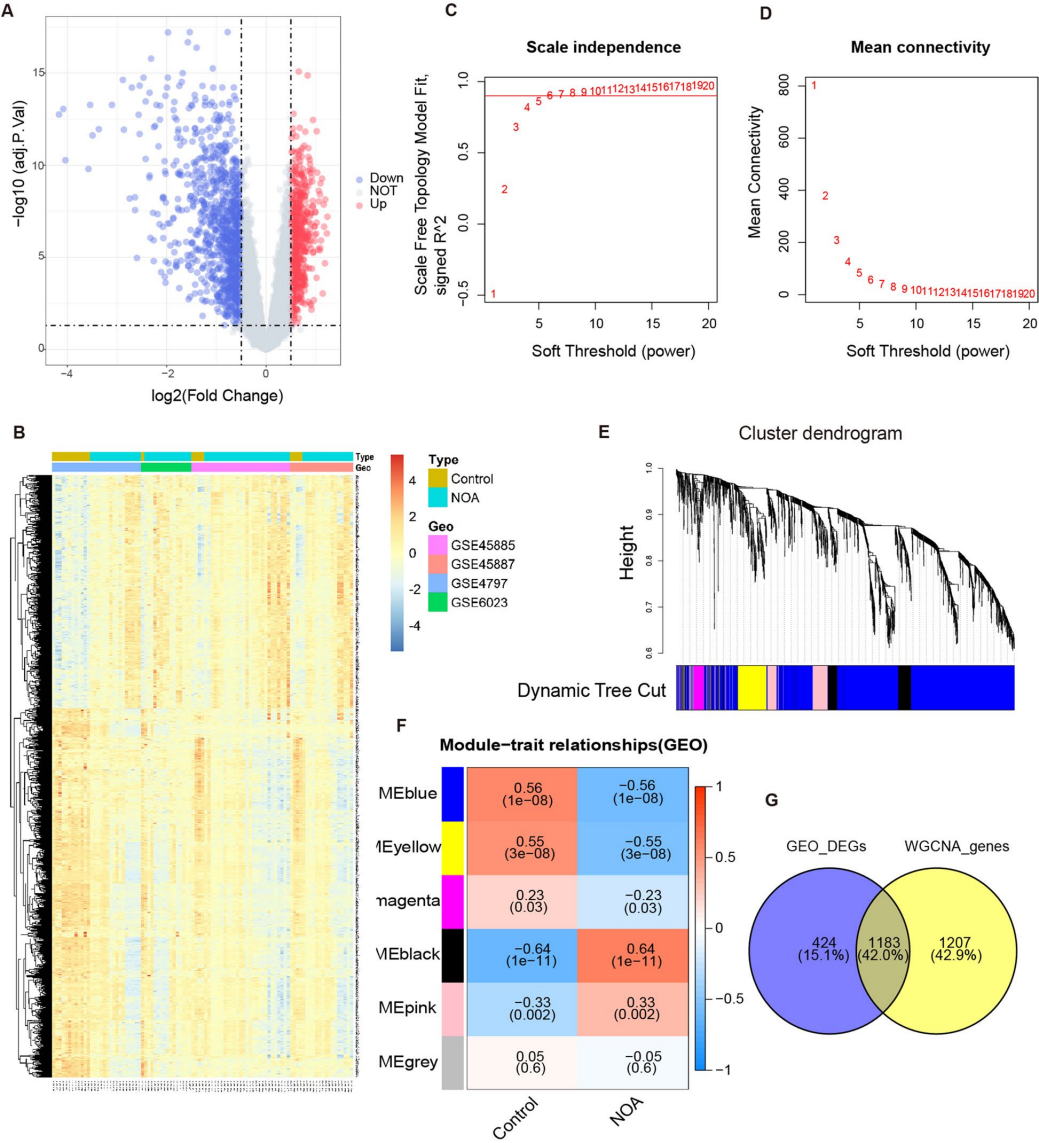
Differentially expressed genes based on integrated analysis and module-trait relationships based on WGCNA analysis in the integrated four GEO datasets (GSE497, GSE6023, GSE45885, GSE45887). **A** The volcano plot of differentially expressed genes between the Control and NOA groups, blue nodes denote down-regulation in NOA; red nodes denote up-regulation, and grey nodes denote no significant difference. **B** The heatmap of differentially expressed genes between the Control and NOA groups. **C** The correlation between scale free topology fit model index (scale free R^2^) and soft threshold. The red horizontal line denotes R^2^ = 0.9. **D** The correlation between mean connectivity and soft threshold. **E** Cluster tree of coexpression modules based on topological overlap measure. **F** Module-trait relationships between the Control and NOA groups based on WGCNA analysis. Different rows (color) denote different gene modules, each column denotes a clinical trait, and each bot shows the correlation coefficient and representative *P* value. **G** Intersection of genes in the GEO_DEGs set and WGCNA_genes set (WGCNA module genes with *P* value < 0.05). The gene count in the middle denotes the genes common to both gene sets. NOA: non-obstructive azoospermia; WGCNA: weighted gene coexpression network analysis. DEGs: differentially expressed genes

The flow diagram systematically illustrated our study strategy (Fig. 1). After screening and normalization, each matrix was merged with the other matrix continuously. We removed the batch effect from the merged dataset. The integrated training dataset was obtained, including the GSE4797, GSE6023, GSE45885, and GSE45887 matrixes. It included 42 samples in the control group and 131 samples in the NOA group. After variance analysis, we identified a total of 1607 significantly differentially expressed genes, including 622 upregulated and 985 downregulated genes (adjusted *P* < 0.05). The volcano plots of DEGs and the heatmap of expression profile were shown in Fig. 2A and B, respectively.

### Module genes based on WGCNA

WGCNA was performed to screen genes that were more consistent with NOA using the integrated dataset. Based on the optimal soft-power threshold, the scale independence and mean connectivity were obtained (Fig. 2C, D). Accordingly, we obtained a cluster dendrogram and six modules involving NOA (Fig. 2E, F). A total of 2394 significant module genes were identified by WGCNA. The blue, yellow, and magenta modules were negatively associated with the NOA phenotype (*P* < 0.01), while the black and pink modules were positively associated with the NOA phenotype (*P* < 0.01). It is expected that using both integrated analysis and WGCNA will help us find more important genes implicated in spermatogenic disorder. Therefore, we intersected the above two gene sets, and 1183 common DEGs (388 upregulated and 795 downregulated) were identified (Fig. 2G).

### GO function relevant to common DEGs

**Fig. 3:**
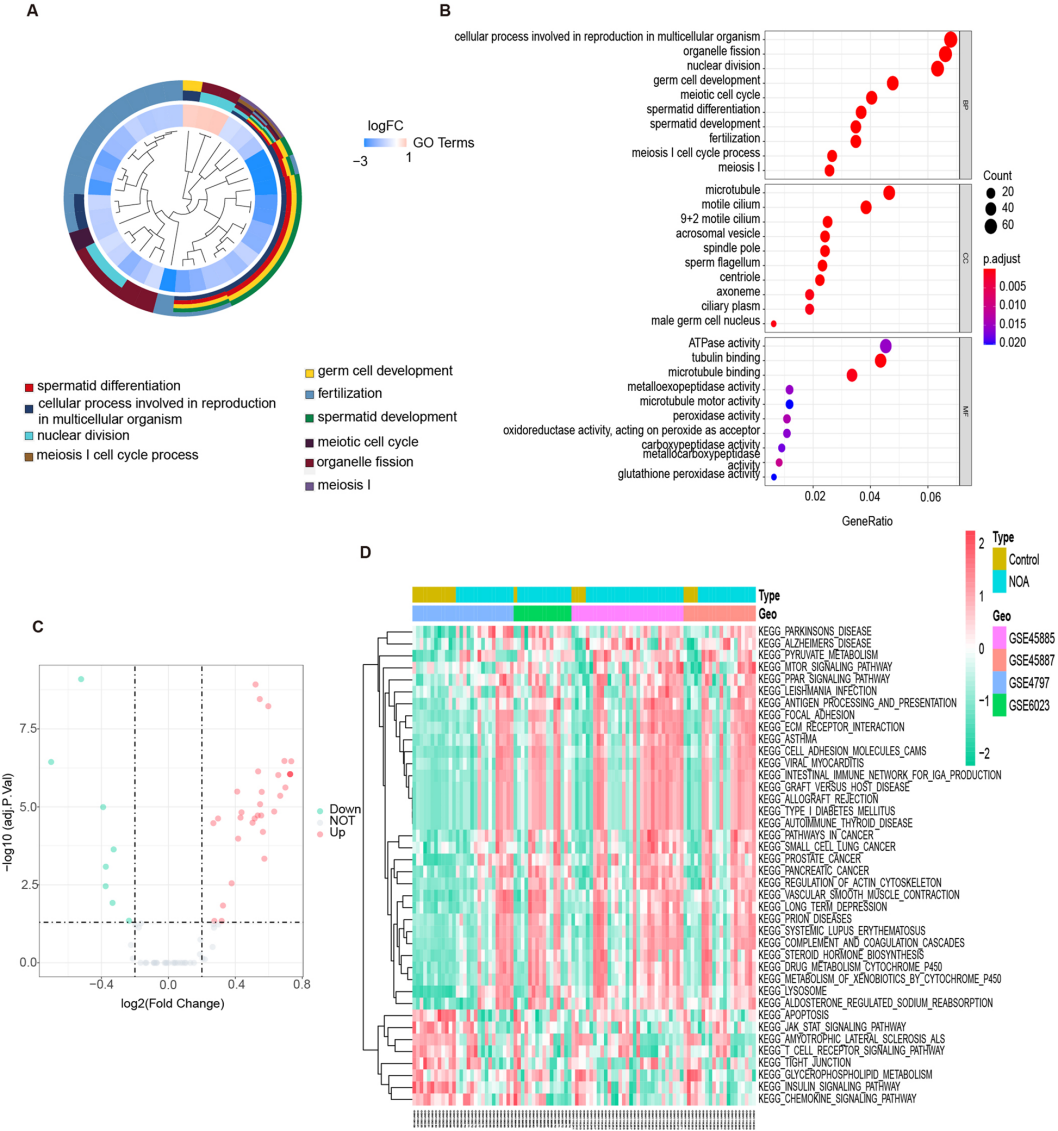
Functional enrichment analysis of common DEGs. **A** GO cluster in common DEGs of NOA patients relative to Control. **B** Enriched GO terms of BP, CC, and MF in common DEGs. **C** The volcano plot of significant KEGG signaling pathways in common DEGs between the Control and NOA groups, as quantified by GSVA. **D** The heatmap of significant signaling pathways between the Control and NOA groups, as quantified by GSVA. NOA: non-obstructive azoospermia; DEGs: differentially expressed genes; BP: biological process; CC: cellular component; MF: molecular function; GSVA: gene set variation analysis

GO analysis was performed to explore the biological function of the common DEGs of NOA. One hundred and thirty-one GO terms were significantly relevant to common DEGs (*P.adjust* < 0.05) (Supplementary Table 5). The top three GO terms in the biological process category involved cellular processes involved in reproduction in multicellular organism, organelle fission, and nuclear division. The top three cellular components involved microtubule, motile cilium, and 9 + 2 motile cilium. The top three in molecular function involved ATPase activity, tubulin binding, and microtubule binding. This finding indicated that the significant biological functions were focused on energy supply and cytoskeleton construction during sperm development (Fig. 3A, B).

### KEGG pathway based on GSVA

GSVA-KEGG pathway analysis was performed to visually display the changes in KEGG pathways. Forty significantly different KEGG pathways were shown in the volcano plot and heatmap (*P.adjust* < 0.05, Fig. 3C, D) (Supplementary Table 6). We found thirty-two significantly upregulated and eight downregulated KEGG pathways in the NOA group compared with the control group. Upregulated pathways were mainly enriched in steroid hormone biosynthesis, pyruvate metabolism, systemic lupus erythematosus, and autoimmune thyroid disease pathway, while downregulated pathways were mainly enriched in JAK-STAT signaling, tight junction, insulin signaling, and glycerophospholipid metabolism pathways. The data suggested that the enriched KEGG pathways for NOA were significantly relevant to metabolic or immune disorders.

### PPI network and key modules

**Fig. 4:**
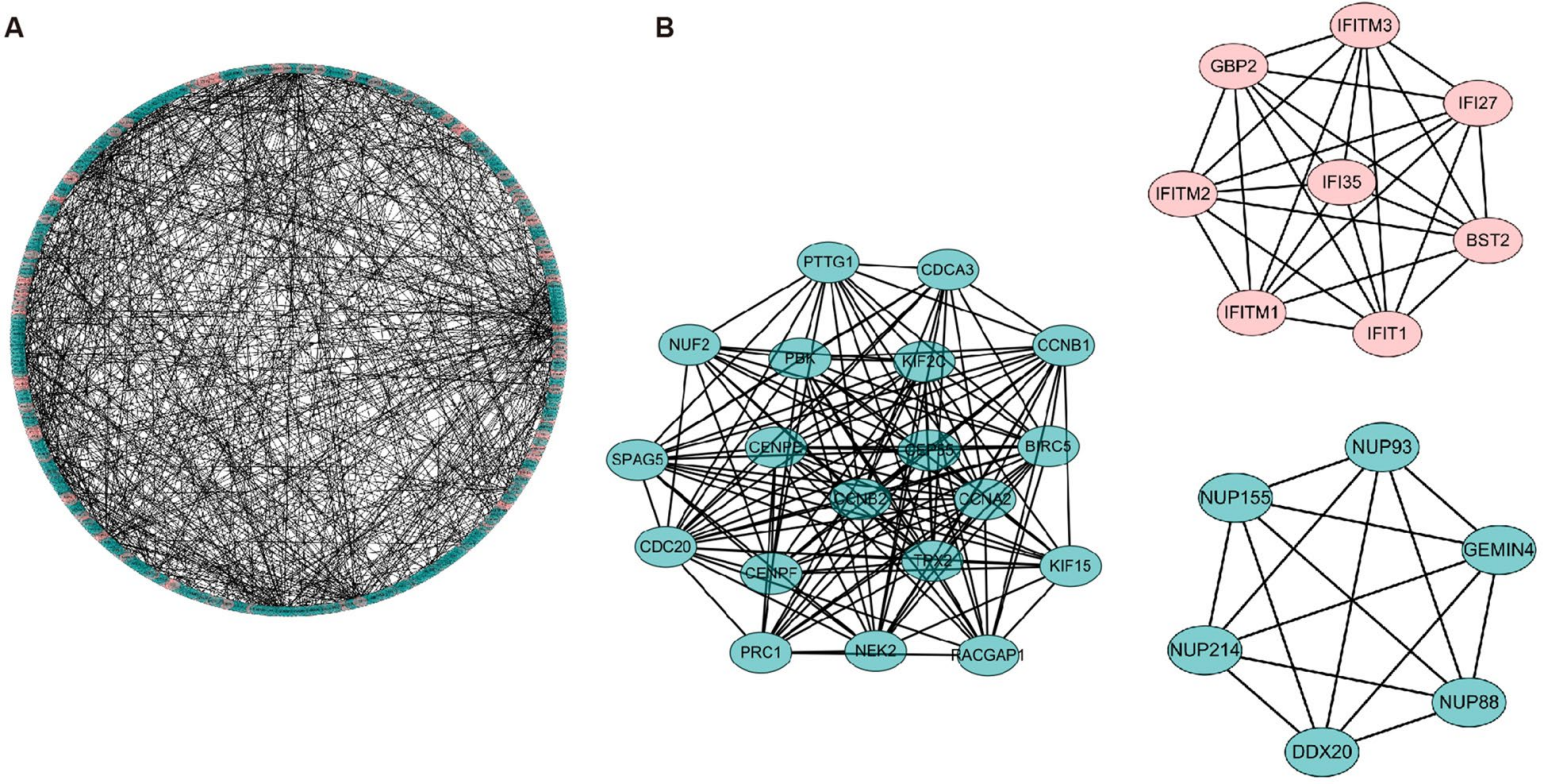
Protein–protein interaction network of common DEGs. **A** The protein–protein (PPI) network showed the interaction of 1183 common DEGs. **B** The three key modules consist of co-expressing common DEGs. For **A** and **B**, red circles denote up-regulated common DEGs, and green circles denote down-regulated ones

To reveal the coexpression profile associated with NOA, the common DEGs were analyzed using the STRING database. The protein–protein interaction (PPI) network included 443 nodes and 815 interactions (Fig. 4A), and the detailed interaction relationship is shown in Supplementary Material 1. Three key coexpression modules were identified by analyzing the Molecular Complex Detection (MCODE) plug-in of cytoHubba (Fig. 4B). The two inhibitory key modules (green) consisted of 19 genes and six genes, respectively, and the activated key module (red) consisted of eight genes.

### External validation of the LASSO model

**Fig. 5:**
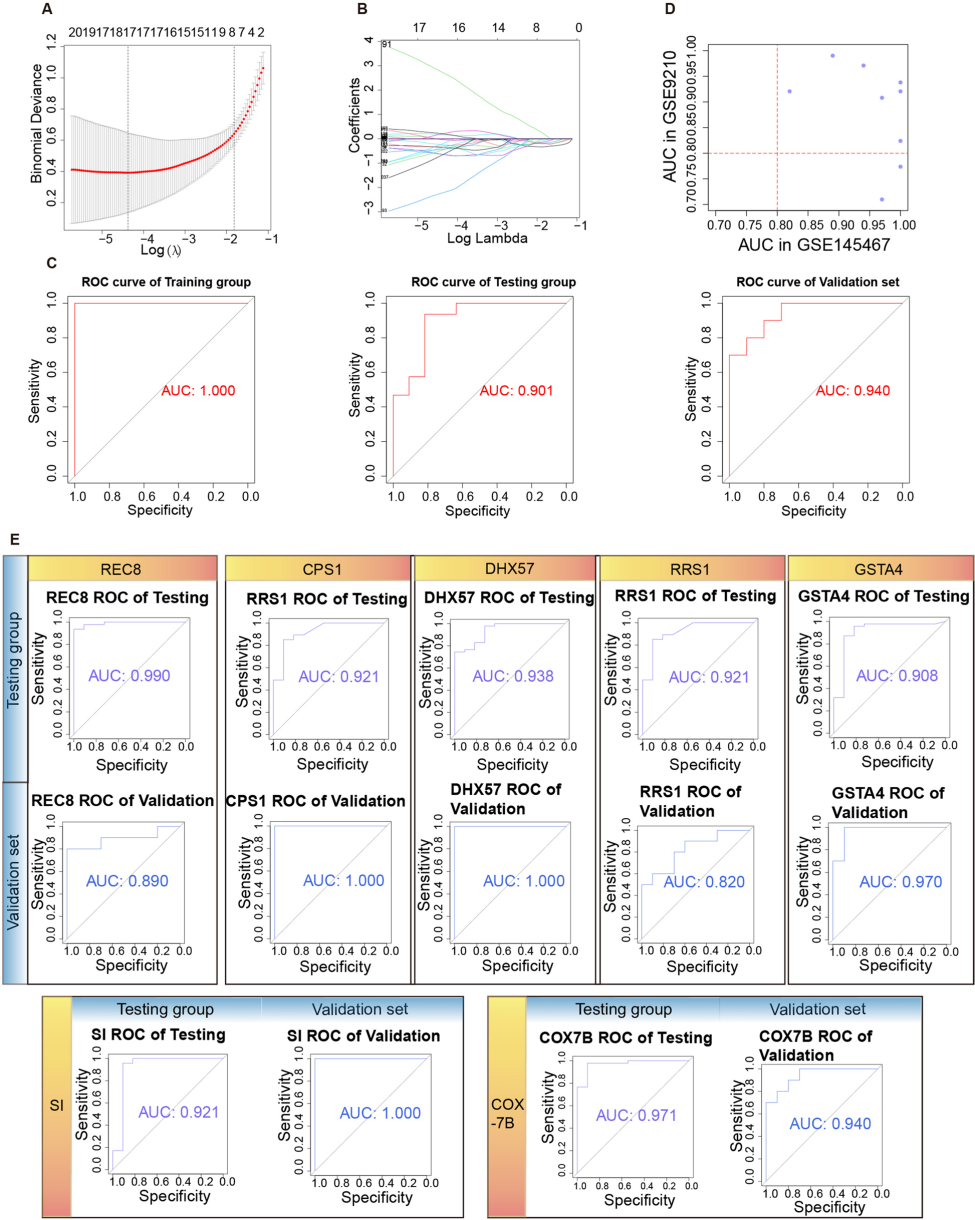
Predictive value of LASSO genes for NOA. **A** The relationship between binomial deviance showed the optimal Lamba parameter. **B** The relationship between LASSO coefficients and turning parameter, each curve denotes a coefficient. **C** The ROC curves of the LASSO model in the training group, testing group, and validation set. **D** The seven LASSO genes with AUCs > 0.8 in the GSE9210 and GSE145467 datasets. **E** The AUCs were shown in the testing group and validation set for the seven key genes, including REC8, CPS1, DHX57, RRS1, GSTA4, SI, and COX7B. NOA: non-obstructive azoospermia; LASSO: Least absolute shrinkage and selection operator; ROC: receiver operating characteristic; AUC: area under the curve

For the predictive value of common DEGs, LASSO regression methodology shows good performance for a large number of covariates among samples [21]. Before obtaining the model, we calculated the binomial deviance and coefficient, as shown in Fig. 5A and B. Finally, seventeen common DEGs were selected, and the risk score was analyzed according to their gene expression level and corresponding regression coefficient. The LASSO model was as follows: risk score = (-0.63901 × Exp of REC8) + (-0.46404 × Exp of ATP1A4) + (-0.64008 × Exp of CPS1) + (-0.24097 × Exp of DHX57) + (-0.03503 × Exp of CYLC2) + (2.72764 × Exp of RRS1) + (-2.22172 × Exp of CNTN2) + (0.16477 × Exp of GSTA4) + (-0.01005 × Exp of CYP2C8) + (-0.35781 × Exp of CPNE2) + (-0.30048 × Exp of PHF1) + (-0.43311 × Exp of APOBEC2) + (-0.36567 × Exp of SI) + (0.20195 × Exp of COX7B) + (0.20354 × Exp of LDLR) + (-0.20368 × Exp of WASF1) + (-0.66296 × Exp of EDIL3).

Then, we performed ROC curve analysis to measure the ability of this seventeen-gene signature model to discriminate between NOA and control samples. The area under the ROC curve (AUC) was 1.000 in the integrated training group. In addition, we applied this model to the testing group and validation set to evaluate the discrimination ability. The AUC value also showed that this model had good accuracy (AUC = 0.901 for testing and 0.940 for validation) (Fig. 5C). Furthermore, we measured the discrimination efficacy of a single gene in the seventeen-gene signature. As shown in Fig. 5D, seven key genes were selected based on their AUCs > 0.8. The AUCs of seven key genes in the respective testing and validation sets were as follows (Fig. 5E): REC8 (AUC = 0.990 and 0.890, *P* < 0.001), CPS1 (AUC = 0.921 and 1.000, *P* < 0.001), DHX57 (AUC = 0.938 and 1.000, *P* < 0.001), RRS1 (AUC = 0.921 and 0.820, *P* < 0.001), GSTA4 (AUC = 0.908 and 0.970, *P* < 0.001), SI (AUC = 0.921 and 1.000, *P* < 0.001), and COX7B (AUC = 0.971 and 0.940, *P* < 0.001).

### Cellular expression profile of seven key genes

**Fig. 6:**
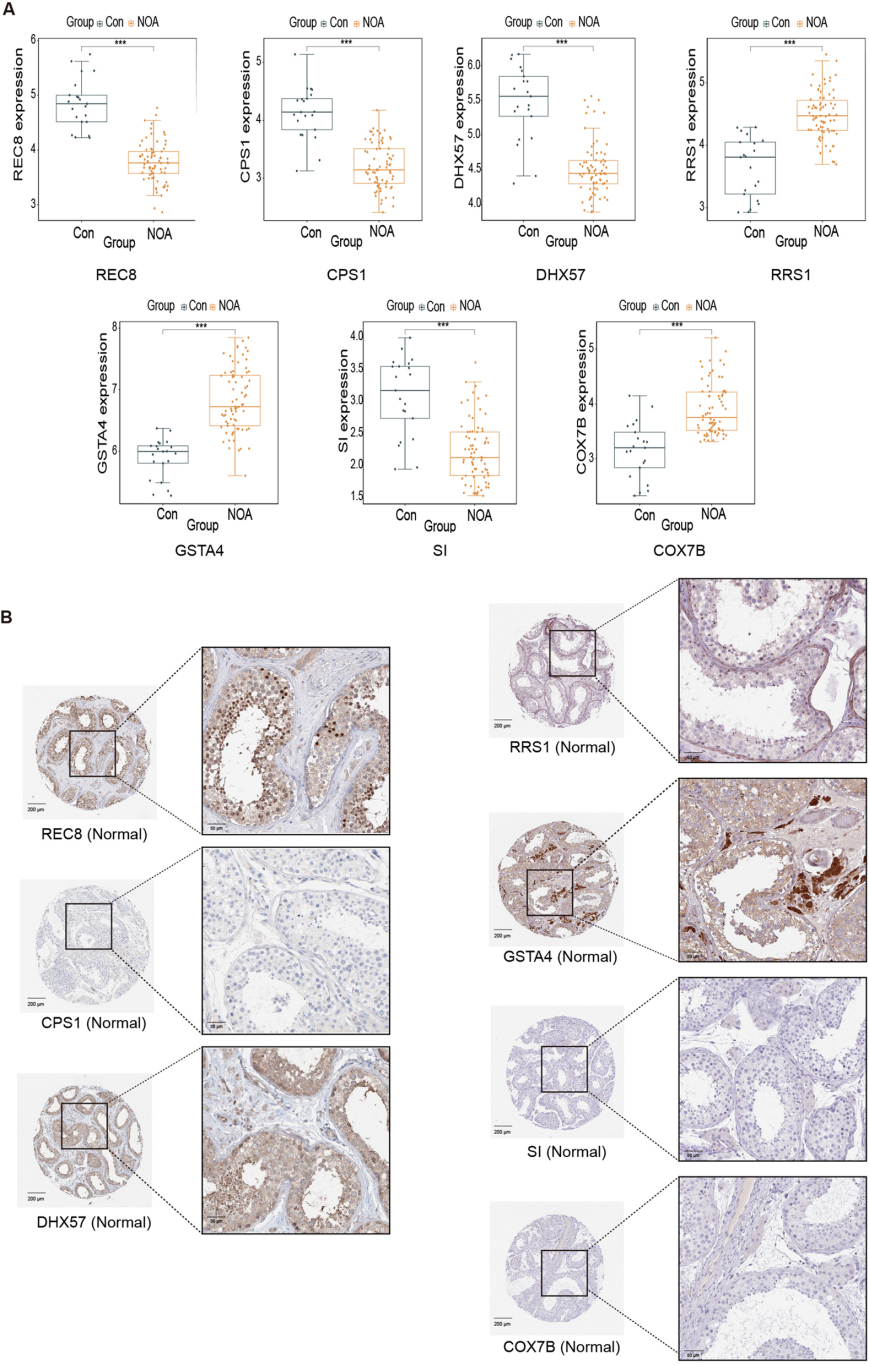
Expression evaluation and subcellular localization of seven key genes. **A** REC8, CPS1, DHX57, RRS1, GSTA4, SI, and COX7B expression between the NOA and Control group. **B** Immunohistochemistry results in the expression of the seven key genes for men with normal fertility in the HPA database. NOA: non-obstructive azoospermia; HPA: Human Protein Atlas

Aside from ROC analysis, expression analysis of the above seven key genes was evaluated in the integrated dataset, immunohistochemical data, and single-cell transcriptome data of the HPA database. We found that the expression of REC8, CPS1, DHX57, and SI significantly decreased in the NOA samples, whereas RRS1, GSTA4, and COX7B expression significantly increased in the NOA samples (Fig. 6A). As shown in Fig. 6B, there was a high positive area of REC8 in round/early spermatids and elongated/late spermatids in healthy testicular tissue, while REC8 expression was not detected in spermatogonia cells. DHX57 was moderately expressed in seminiferous ducts and Leydig cells. RRS1 expression was enriched in Sertoli cells and Leydig cells. GSTA4 was enriched in Leydig cells, and it was expressed at a low level in spermatocytes and round/early spermatids. CPS1, SI, and COX7B were hard to detect in the seminiferous ducts, peritubular cells and Leydig cells.

**Fig. 7:**
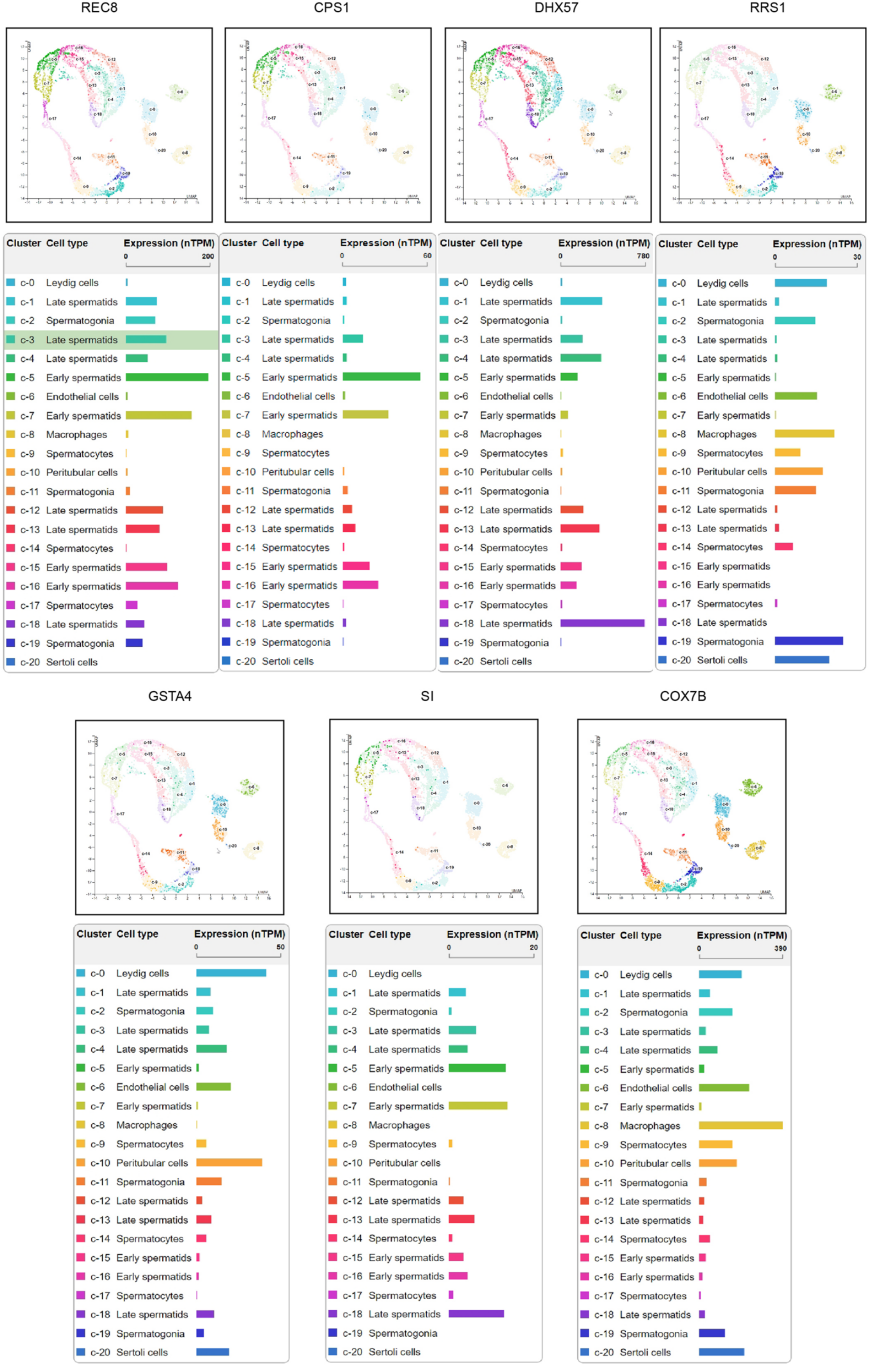
Single-cell transcriptome analysis of the seven key genes in the HPA database. NOA: non-obstructive azoospermia; HPA: Human Protein Atlas

Subsequently, we analyzed the single-cell expression pattern and the developmental trajectory from the HPA database. As shown in Fig. 7, REC8 was maintained at a low level in the clusters of spermatogonia and spermatocyte, and it began to rise in the clusters of early/late spermatids. In addition to being expressed at a low level in all clusters, CPS1 and SI gradually increased in the early spermatids, while their levels decreased to some degree during the late spermatid period. Similarly, DHX57 increased in the early differentiation clusters of late spermatids. However, the peak of RRS1 expression was in the early spermatogonia clusters and decreased during the spermatocyte period. GSTA4 was expressed at low levels in the Leydig cells and peritubular cells. During the transition period from spermatogonia to spermatocyte, COX7B showed medium expression levels in these clusters together with macrophages but decreased expression in late spermatocytes. The results indicated that these seven key genes might play an important role in spermatogenesis with their unique temporal and spatial characteristics.

### Clinical characteristics of patients

We recruited six patients (including three with NOA and three with OA) and collected their testicular samples. The three NOA patients had normal somatic karyotypes and no Y-chromosome microdeletions. Decreased testicular volumes and increased FSH levels were observed in these three NOA patients. Patient 2 with NOA had a low level of serum testosterone. No spermatogenic cells or sperm were seen in the testicular biopsy samples. The three OA patients had decreased levels of neutral alpha-glucosidase and/or increased levels of elastase, indicating obstruction in the epididymis/vas deferens/ejaculatory duct. No obvious sex hormone disorder was observed in those patients. However, spermatids and sperm were observed in the testicular biopsies (Supplementary Table 7).

### Expression validation through qRT-PCR

**Fig. 8:**
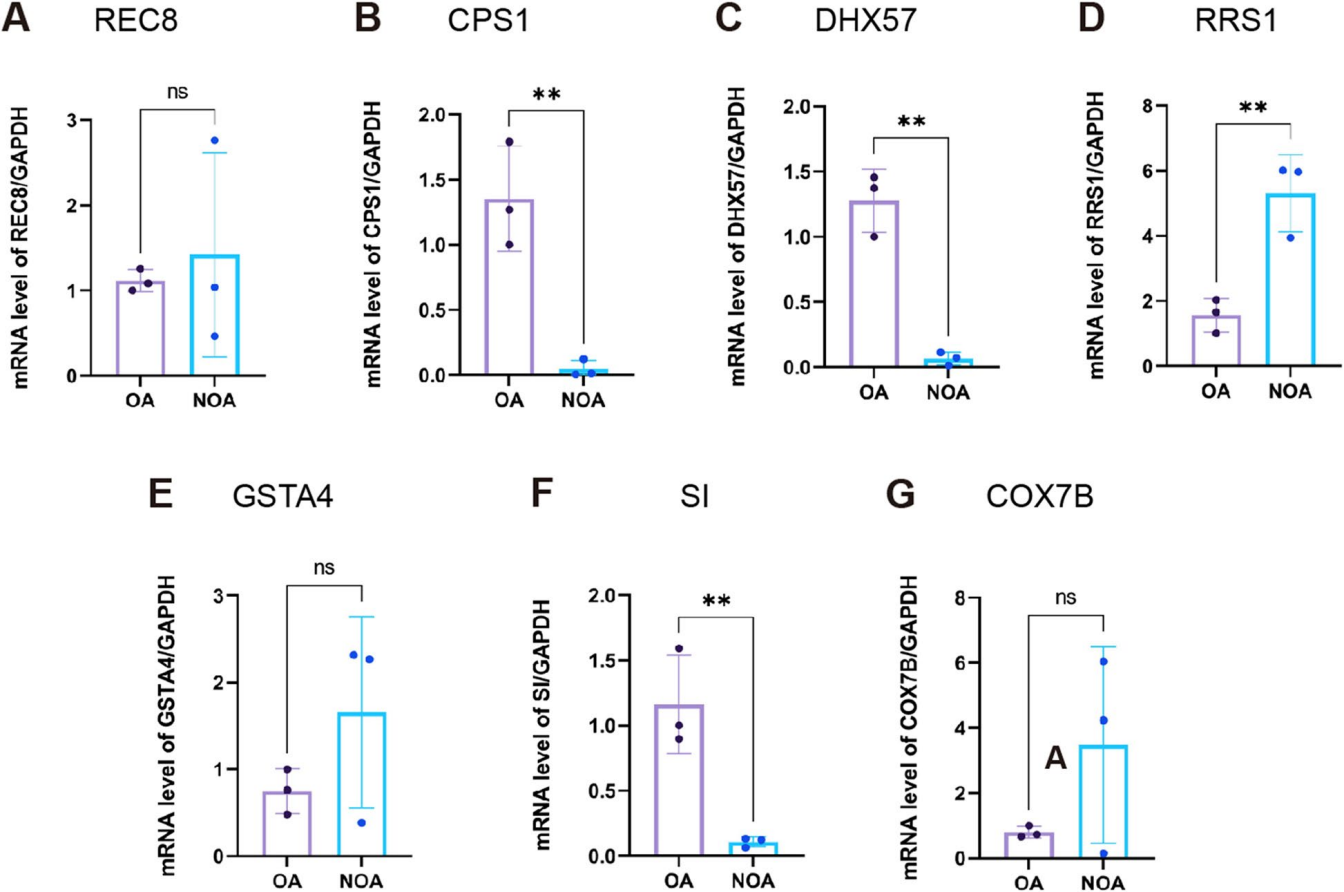
The mRNA levels of seven key genes in the testicular tissues from patients with NOA and OA. mRNA levels of REC8 (**A**), CPS1 (**B**), DHX57 (**C**), RRS1 (**D**), GSTA4 (**E**), SI (**F**) and COX7B (**G**) with GAPDH as the loading control in the two groups. ** denotes *P* < 0.01, NOA: non-obstructive azoospermia; OA: obstructive azoospermia; ns: not significant

We selected seven key genes with AUC > 0.8 to determine their mRNA levels in the testicular tissues from patients with NOA and OA (controls). Consistent with the sequencing data, the mRNA levels of three genes (RRS1, GSTA4, and COX7B) were upregulated, while the other three (CPS1, DHX57, and SI) were downregulated in NOA samples (Fig. 8). The mRNA level of REC8 showed no tendency. There were statistically significant differences in the mRNA levels of CPS1, DHX57, RRS1, and SI. CPS1 (*P* < 0.01), DHX57 (*P* < 0.01), and SI (*P* < 0.01) were expressed at low levels in NOA tissues compared with normal spermatogenesis tissues, while RRS1 (*P* < 0.01) was highly expressed in NOA tissues compared with normal spermatogenesis tissues.

## Discussion

Male infertility is an important problem of human reproductive health in today's society. Over the past 40 years, male fertility has declined globally (1973–2011) [30]. Compared to patients with obstructive azoospermia, who have normal spermatogenesis, patients with NOA often exhibit spermatogenic disorders of different types and degrees. At least three subtypes of histology were included: (a) hypospermatogenesis, (b) maturation arrest (MA) of germ cells (e.g., pre/meiotic arrest), and (c) Sertoli cell-only syndrome (SCOS) [7]. Researchers have reported that numerous anomalies in genetic structure and function are linked to spermatogenic disorders [31]. Therefore, it is of significant value to identify key genes in NOA, not only to elucidate the underlying molecular mechanisms, but also to find potential therapeutic targets for NOA.

In the previous bioinformatic literature regarding male infertility [32–34], differentially expressed gene analysis was widely used in the data processing of microarray or transcriptome data to find robust significant genes. In the above literature, DEG analysis was used to identify important genes from one or two GEO datasets and small sample sizes. In addition, some researchers have used WGCNA alone to find significant genes [35]. However, their results were not validated in other independent datasets. In the present study, to maximize the discovery of potential key genes, we used three bioinformatic methods to find NOA-associated genes from the training group, namely, integrated analysis, WGCNA, and LASSO regression. A seventeen-gene signature predictive model was constructed, and we further verified its predictive performance in the other two independent datasets (GSE9210 and GSE145467). Multilayer screening methodology will help us to obtain DEGs that are more specific to NOA and to explore the underlying etiology.

Herein, 388 upregulated and 795 downregulated common DEGs (after the intersection) were identified. Moreover, we realized that more than half of the DEGs were downregulated in NOA. Previously, three bioinformatics studies on testicular dysfunction revealed that the number of downregulated DEGs was 1.6 to 5.9 times greater than that of upregulated DEGs [32, 35, 36]. Based on the results of DEG analysis in the present study, it is legitimate to speculate that the inhibition of numerous genes in the testes might be responsible for the occurrence and development of NOA. With the rapid development of single-cell RNA sequencing technology, researchers can better explore the gene function of different cell populations in the testicular tissues of NOA patients. Similarly, Mei et al. found that in 174 individual cells from an NOA patient, more genes were downregulated in both Sertoli and Leydig/peritubular myoid cells compared with upregulated genes [37].

GO enrichment analysis revealed that the common DEGs were significantly related to ATPase activity, tubulin binding, and microtubule binding. Ion-exchanging ATPase is not only required for energy metabolism in spermatogenesis but also for sperm maturation. For example, an evident reduction in Na^+^, K^+^-ATPase activity was related to asthenozoospermia [38]. A in vivo study also found that alteration in vacuolar-ATPase activity could disturb the process of sperm maturation and the balance of apoptosis, which may lead to male infertility [39]. Tubulin is the main component of microtubules, which are required for sperm production and maturation [40]. Abnormalities in microtubule binding may cause segregation errors, subsequently leading to aneuploidies [41]. Furthermore, an in vitro study also confirmed that the mutations identified in patients with SCOS are obviously associated with impaired microtubule formation [42]. Given the aforementioned segregation errors, under the condition wherein tubulin and microtubule binding signals were destroyed, it is likely that cell division of spermatogonia and spermatocytes is severely hindered in NOA.

With respect to significant signaling, GSVA-KEGG analysis revealed that JAK_STAT signaling was significantly downregulated in NOA. Current evidence shows that JAK-STAT signaling plays a role in the transformation of spermatogonial stem cells (SSCs) into differentiating spermatogonia [43]. Moreover, researchers also found that members of the JAK/STAT protein family are expressed at the apical region of the sperm head, and confirmed that they are enriched in sperm membrane fractions and cytoskeletal structures [44]. Combined with the above findings, one of the underlying mechanisms for NOA may be linked to JAK-STAT-induced dysregulation in SSCs and sperm formation. For the upregulated signaling pathways, we found that they were mainly enriched in the signaling pathways of glucose metabolism (pyruvate metabolism, insulin signaling, glycerophospholipid metabolism) and immune response (autoimmune thyroid disease and systemic lupus erythematosus). This means that many signaling molecules are shared with spermatogenesis in the above pathway. This further suggests that clinicians need to pay more attention to the possible metabolic and immunological risks associated with male infertility.

Among these seventeen genes in the predictive model, seven genes with higher predictive values were selected (AUC > 0.8). Among the downregulated key genes, *REC8* encodes a key component of the meiotic cohesin complex. It is regarded as a candidate gene for male infertility, and has a vital role in homologous chromosome synapsis [45, 46]. An in vivo study found that primary spermatocytes were absent in the seminiferous tubules of *REC8* null mice [46]. Recent research showed that a patient with NOA harbored a loss-of-function frameshift mutation (p.Pro287ArgfsTer74) in the *REC8* gene. Unfortunately, the patient’s outcome of testicular sperm extraction was negative [47]. Notably, the expression pattern of REC8 has spatiotemporal specificity. It is expressed at a relatively low level in spermatocytes but is highly expressed in early/late spermatids.

Herein, we found several new downregulated key genes in NOA, but they are rarely reported in spermatogenesis. CPS1 (carbamoyl phosphate synthetase 1) acts as the first and rate-limiting urea cycle enzyme that converts ammonia to carbamoyl phosphate and is well known for its role in malignant disease [48, 49]. CPS1 deficiency can lead to refusal to eat, coma, hypotonia, convulsions, lethargy, and even death [50]. However, investigation on its relation to fertility is limited. One study reported CPS1 expression in human testes in adults compared with fetuses [51]. Another study showed that CPS1, acting as a SIRT5 target, was responsible for an accumulation of follicular-fluid ammonium in women with reduced ovarian reserve or advanced maternal age [52]. Most evidence has focused on gastrointestinal tract function and cellular RNA metabolism regarding the *SI* and *DHX57* genes, respectively. For example, a deficiency of the *SI* gene contributes to carbohydrate malabsorption characterized by abdominal pain, bloating, watery, and osmotic-fermentative diarrhea [53]. DExH-box helicases serve as one of the main families of RNA helicases and generally have sequence-independent but structure-dependent characteristics [54]. Nonetheless, the functionality of these two genes in spermatogenesis is unclear. Our study found that the expression of SI (sucrase-isomaltase) and DHX57 (DExH-box helicase 57) was reported in healthy testis, but the expression of both genes decreased significantly in NOA patients, which might indicate abnormalities in glycometabolism and RNA metabolism in spermatogenic failure.

In contrast, three key genes were identified to be upregulated in NOA. RRS1 (regulator of ribosome synthesis 1) is principally localized in the nucleolus and endoplasmic reticulum [55]. Recent studies regarded RRS1 as a tumor promotor and concluded that overexpression of RRS1 could facilitate the growth and metastasis of various tumors [56]. In the molecular mechanism, RRS1 participates in ribosome biosynthesis and mainly influences chromosomal and telomeric arrangement during the cell cycle [57]. In Fig. 6, RRS1 is mainly expressed in the early cluster of spermatogonia, indicating that defects in this gene may disturb the mitosis of spermatogonia. GSTA4 belongs to the enzymes implicated as cellular guards against carcinogenic, toxic, and pharmacologically active electrophilic damage. Consistent with our single-cell expression pattern of GSTA4 in Leydig cells, GSTA4 activity is involved in the steroidogenesis of Leydig cells [58]. Zhang et al. also found that abnormalities in sperm quality and quantity were evidently associated with GSTA4 upregulation in a mouse model [59]. Our study identified aberrant overexpression of GSTA4 in the NOA group. These findings indicated that increased GSTA4 activity may affect testicular function possibly via disturbance of Leydig cells. *COX7B* encodes a structural subunit of cytochrome c oxidase that regulates COX assembly and mitochondrial respiration [60]. A relationship between mitochondrial respiration and spermatogonia differentiation was identified. A recent study found that impaired mitochondrial structure and function were positively associated with enhanced autophagy, as well as higher apoptosis and necrosis in spermatogonia [61]. Notably, we found a high predictive value of COX7B in NOA, suggesting that COX7-mediated mitochondrial respiratory dysfunction may play an important role in the evolution of spermatogenic failure.

To verify our sequencing results, we provided immunohistochemical data, and single-cell transcriptome data from the HPA database and performed qRT-PCR for seven key genes in testicular tissues of both the NOA and control groups. The data showed that consistent with the integrated microarray results, the mRNA levels of RRS1, GSTA4, and COX7B were upregulated, while those of CPS1, DHX57, and SI were downregulated in NOA. Only four genes (CPS1, DHX57, RRS1, and SI) showed statistically significant differences. Although the mRNA levels of the other two genes (GSTA4 and COX7B) were different between the NOA and control groups, the differences did not reach statistically significant (*P* < 0.25). The reasons underlying the gap between the microarray and qRT-PCR results might be as follows: 1) the present sample size was not large enough (*n* = 6), and this may further affect the significance of difference between the groups; 2) there may be heterogeneity in the histology of testes from different patients, and the heterogeneity may result in an insignificant tendency in some genes.

As a severe type of male infertility, the treatment of NOA has always troubled reproductive physicians and andrologists due to limited regimens and poor therapeutic effects. Even when utilizing micro-TESE, the success rate is only approximately 50%. The success rate may be lower if testicular sperm aspiration is employed, but the expense will be significantly reduced. Therefore, early detection, early diagnosis and early treatment for NOA are particularly important. Using the dynamic genetic model might have great potential to achieve this goal. This means that we may not only help NOA patients preserve fertility as early as possible but may also help them predict the outcome of sperm retrieval and further choose a more cost-effective assisted reproductive technology. In our study, we obtained a seventeen-gene signature model and identified seven key genes with significant predictive values. These key genes might be useful for the better understanding of pathogenesis and may become potential targets for pharmaceutical discovery. However, this study has several limitations. First, although the predictive model was validated in two independent datasets, experimental validation was performed in six testicular samples. It is of great importance to collect more samples to conduct experimental validation, and the NOA samples should include three subtypes of histology for validation. There is a need to confirm the efficacy in a larger cohort and to perform a prospective study. Second, the bio-omics data come from different races and regions. Differences in clinical data may affect the reliability of LASSO analysis. Third, the specific function of the seven key genes in the spermatogenic environment was not well explored, and in-depth molecular investigation is needed.

## Conclusions

In summary, we used a total of four microarray datasets to establish a risk score model for NOA patients with seventeen genes and further validated the efficacy of the model in two additional independent datasets. Seven key genes with good predictive values were further selected and characterized for their expression level, localization and developmental trajectory. Based on the value of the risk model and key genes, it would be helpful for clinicians to better understand the pathogenesis of NOA. In the meantime, these seven key genes with high clinical specificity to NOA are worth exploring in depth. Whether as an important regulator of the pathogenesis of male infertility, or as a potential drug target, their research value and prospects are significant and cannot be ignored.

## Supplementary Information

**Additional file 1:** **Supplementary Figure 1.** Normalization processing and analysis of six GEO datasets (GSE4797, GSE6023, GSE45885, GSE45887, GSE9210, and GSE145467).

**Additional file 2:** **Supplementary Table 1.** Six GEO datasets used in this study.

**Additional file 3:** **Supplementary Table 2.** Immunohistochemical data of seven key genes in the HPA database.

**Additional file 4:** **Supplementary Table 3.** Single-cell transcriptome data of seven key genes in the HPA database.

**Additional file 5:** **Supplementary Table 4.** The oligonucleotide sequences of primers used in quantitative real-time PCR.

**Additional file 6:** **Supplementary Table 5.** GO enrichment analysis of common DEGs.

**Additional file 7:** **Supplementary Table 6.** KEGG enrichment analysis of common DEGs by GSVA.

**Additional file 8:** **Supplementary Table 7.** Clinical characteristics of patients with NOA and OA.

**Additional file 9:** **Supplementary Material 1.** Detailed information of protein-protein interaction network.

## Data Availability

The datasets generated and/or analyzed during the current study are available in the GEO (http://www.ncbi.nlm.nih.gov/geo) repository and the HPA repository (https://www.proteinatlas.org/). [(GSE4797) (https://www.ncbi.nlm.nih.gov/geo/query/acc.cgi?acc=GSE4797)], [(GSE6023) (https://www.ncbi.nlm.nih.gov/geo/query/acc.cgi?acc=GSE6023)], [(GSE45885) (https://www.ncbi.nlm.nih.gov/geo/query/acc.cgi?acc=GSE45885)], [(GSE45887) (https://www.ncbi.nlm.nih.gov/geo/query/acc.cgi?acc=GSE45887)], [(GSE9210) (https://www.ncbi.nlm.nih.gov/geo/query/acc.cgi?acc=GSE9210)], [(GSE145467) (https://www.ncbi.nlm.nih.gov/geo/query/acc.cgi?acc=GSE145467)].

## Abbreviations

NOA: Non-obstructive azoospermia
GEO: Gene Expression Omnibus
WGCNA: Weighted correlation network analysis
DESs: Differentially expressed genes
LASSO: Least Absolute Shrinkage and Selection Operation
qRT-PCR: Quantitative real-time polymerase chain reaction
HPA: Human Protein Atlas
ROC: Receiver operating characteristic
AUC: Areas under the curve
TESE: Testicular sperm extraction
EAU: European Association of Urology
SNP: Single nucleotide polymorphism
GWAS: Genome-wide association studies
log2FC: Log2 fold-change
TOM: Topological overlap measure
GO: Gene Ontology
PPI: Protein–protein interaction
GSVA: Gene set variation analysis
KEGG: Kyoto Encyclopedia of Genes and Genomes
SD: Standard deviation
MCODE: Molecular Complex Detection
MA: Maturation arrest
SCOS: Sertoli-Cell-Only Syndrome
