# Supplementary figures and images for: Constructing a seventeen-gene signature model for non-obstructive azoospermia based on integrated transcriptome analyses and WGCNA
Source: Reprod Biol Endocrinol. 2023 Mar 21;21:30. doi: 10.1186/s12958-023-01079-5 (PMC10029246; doi:10.1186/s12958-023-01079-5)

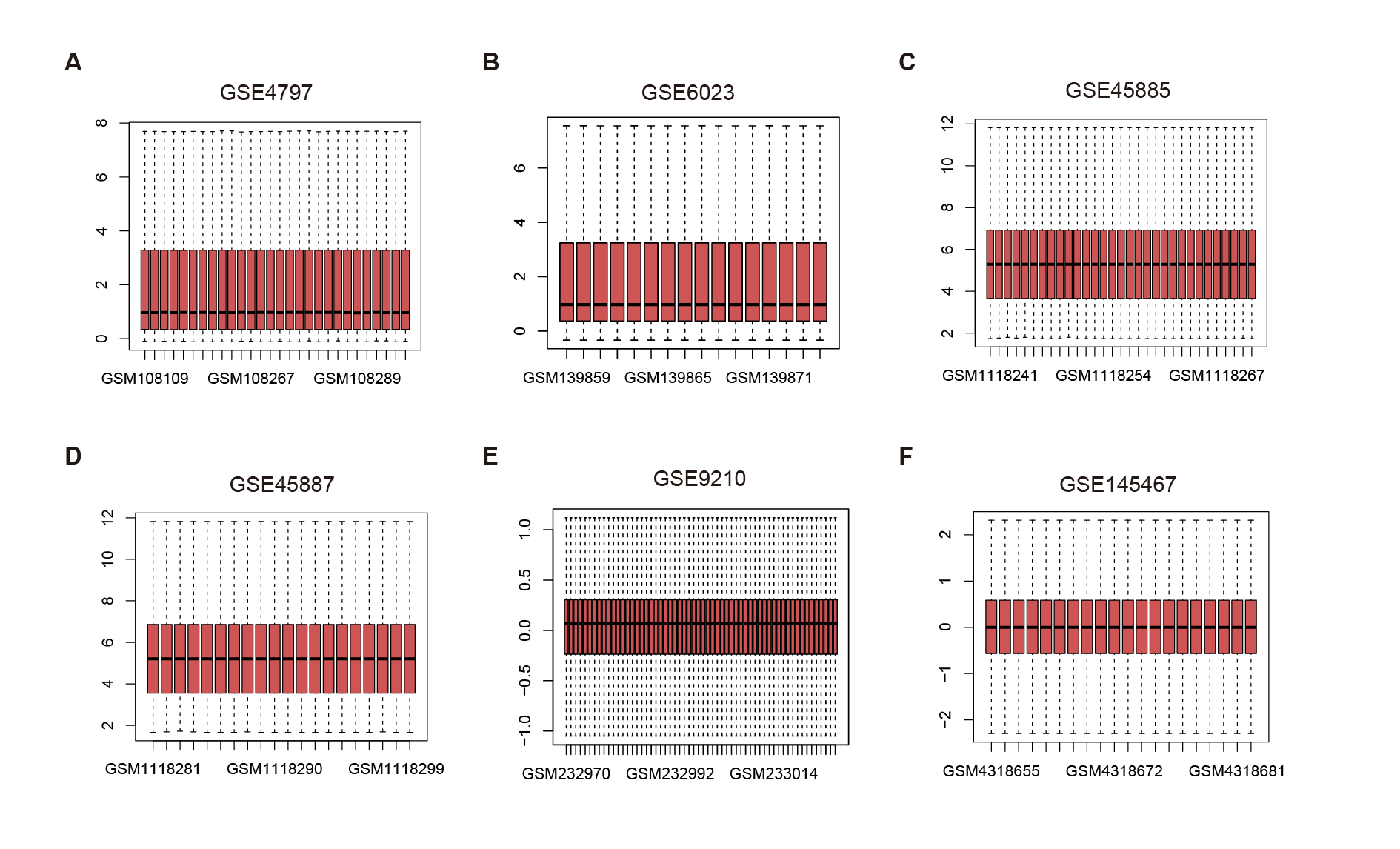

Supplement: Supplementary file 1 — Additional file 1: Supplementary Figure 1. Normalization processing and analysis of six GEO datasets (GSE4797, GSE6023, GSE45885, GSE45887, GSE9210, and GSE145467). [file 12958_2023_1079_MOESM1_ESM.jpg]
