# Supplementary material for: Constructing a seventeen-gene signature model for non-obstructive azoospermia based on integrated transcriptome analyses and WGCNA
Source: Reprod Biol Endocrinol. 2023 Mar 21;21:30. doi: 10.1186/s12958-023-01079-5 (PMC10029246; doi:10.1186/s12958-023-01079-5)
Supplement: Supplementary file 2 — Additional file 2: Supplementary Table 1. Six GEO datasets used in this study. [file 12958_2023_1079_MOESM2_ESM.docx]

**Supplementary Table 1**

Six GEO datasets used in this study.

| Dataset | Numbers of samples  (normal spermatogenesis  /NOA) | Platform | Last update date | Country |
| --- | --- | --- | --- | --- |
| GSE4797 | 28(12/16) | GPL2891 | Sep 27, 2013 | Germany |
| GSE6023 | 16(1/15) | GPL2891 | Sep 27, 2013 | Germany |
| GSE45885 | 31(4/27) | GPL6244 | Jul 26, 2018 | Norway |
| GSE45887 | 20(4/16) | GPL6244 | Jul 26, 2018 | Norway |
| GSE9210 | 58(11/47) | GPL887 | Dec 06, 2012 | Japan |
| GSE145467 | 20(10/10) | GPL4133 | Feb 23, 2020 | Slovenia |

GEO: Gene Expression Omnibus; GSE: GEO Series; GPL: GEO Platform; NOA: non-obstructive azoospermia.
