# Supplementary material for: Constructing a seventeen-gene signature model for non-obstructive azoospermia based on integrated transcriptome analyses and WGCNA
Source: Reprod Biol Endocrinol. 2023 Mar 21;21:30. doi: 10.1186/s12958-023-01079-5 (PMC10029246; doi:10.1186/s12958-023-01079-5)
Supplement: Supplementary file 3 — Additional file 3: Supplementary Table 2. Immunohistochemical data of seven key genes in the HPA database. [file 12958_2023_1079_MOESM3_ESM.docx]

**Supplementary Table 2**

Immunohistochemical data of seven key genes in the HPA database.

| Gene | URL | Protein Atlas version |
| --- | --- | --- |
| REC8 | https://www.proteinatlas.org/ENSG00000100918-REC8/tissue/testis#img | Version 21.0 |
| CPS1 | https://www.proteinatlas.org/ENSG00000021826-CPS1/tissue/testis#img | Version 21.0 |
| DHX57 | https://www.proteinatlas.org/ENSG00000163214-DHX57/tissue/testis#img | Version 21.0 |
| RRS1 | https://www.proteinatlas.org/ENSG00000179041-RRS1/tissue/testis#img | Version 21.0 |
| GSTA4 | https://www.proteinatlas.org/ENSG00000170899-GSTA4/tissue/testis#img | Version 21.0 |
| SI | https://www.proteinatlas.org/ENSG00000090402-SI/tissue/testis#img | Version 21.0 |
| COX7B | https://www.proteinatlas.org/ENSG00000131174-COX7B/tissue/testis#img | Version 21.0 |

HPA database: Human Protein Atlas database; URL: uniform resource locator.
