# Supplementary material for: Constructing a seventeen-gene signature model for non-obstructive azoospermia based on integrated transcriptome analyses and WGCNA
Source: Reprod Biol Endocrinol. 2023 Mar 21;21:30. doi: 10.1186/s12958-023-01079-5 (PMC10029246; doi:10.1186/s12958-023-01079-5)
Supplement: Supplementary file 5 — Additional file 5: Supplementary Table 4. The oligonucleotide sequences of primers used in quantitative real-time PCR. [file 12958_2023_1079_MOESM5_ESM.docx]

**Supplementary Table 4**

The oligonucleotide sequences of primers used in quantitative real-time PCR.

| Gene | Primer type | Sequence/Target sequence |
| --- | --- | --- |
| REC8 (human) | Forward | 5’-TCTACTATCCCAACGTGCTTCAG-3’ |
| REC8 (human) | Reverse | 5’-CTGAAGTTGGGCTGAGAGATAGA-3’ |
| CPS1 (human) | Forward | 5’-CAGACAAGGGAGCTGAGGTTAAA-3’ |
| CPS1 (human) | Reverse | 5’-ACATCATGCTCCTGACCATTGTA-3’ |
| DHX57 (human) | Forward | 5’-CTCCTGTGAACTTTCTGCCAGTA-3’ |
| DHX57 (human) | Reverse | 5’-ACTTTGCCTGCCAGTCATATCTT-3’ |
| RRS1 (human) | Forward | 5’-GGGACTTTGCAGCCGAGAAA-3’ |
| RRS1 (human) | Reverse | 5’-TCCTCCTCCCTCATCTGCTTATT-3’ |
| GSTA4 (human) | Forward | 5’-AACAAGTGCCCATGGTTGAAATT-3’ |
| GSTA4 (human) | Reverse | 5’-TGATAAGCAGTTCCAGCAGATCC-3’ |
| SI (human) | Forward | 5’-GGCCGTGGAATGACTCTCTTATT-3’ |
| SI (human) | Reverse | 5’-GGAAACGATTGGGTGTCTGATTT-3’ |
| COX7B (human) | Forward | 5’-TCTAGCTTCACCTTCACGATGTT-3’ |
| COX7B (human) | Reverse | 5’-TCTATTCCGACTTGTGTTGCTACA-3’ |
| GAPDH (human) | Forward | 5’- GGAGCGAGATCCCTCCAAAAT-3’ |
| GAPDH (human) | Reverse | 5’- GGCTGTTGTCATACTTCTCATGG-3’ |

REC8: REC8 meiotic recombination protein; CPS1: carbamoyl-phosphate synthase 1; DHX57: carbamoyl-phosphate synthase 1; RRS1: ribosome biogenesis regulator 1 homolog; GSTA4: glutathione S-transferase alpha 4; SI: sucrase-isomaltase; COX7B: cytochrome c oxidase subunit 7B; GAPDH: glyceraldehyde-3-phosphate dehydrogenase.
