# Supplementary material for: Constructing a seventeen-gene signature model for non-obstructive azoospermia based on integrated transcriptome analyses and WGCNA
Source: Reprod Biol Endocrinol. 2023 Mar 21;21:30. doi: 10.1186/s12958-023-01079-5 (PMC10029246; doi:10.1186/s12958-023-01079-5)
Supplement: Supplementary file 6 — Additional file 6: Supplementary Table 5. GO enrichment analysis of common DEGs. [file 12958_2023_1079_MOESM6_ESM.docx]

**Supplementary Table 5**

GO enrichment analysis of common DEGs.

| Ontology | ID | Description | p.adjust | Count |
| --- | --- | --- | --- | --- |
| BP | GO:0022412 | cellular process involved in reproduction in multicellular organism | 1.23E-18 | 74 |
| BP | GO:0048515 | spermatid differentiation | 4.50E-12 | 40 |
| BP | GO:0007281 | germ cell development | 4.50E-12 | 52 |
| BP | GO:0007286 | spermatid development | 1.77E-11 | 38 |
| BP | GO:0000280 | nuclear division | 1.77E-11 | 69 |
| BP | GO:0048285 | organelle fission | 1.19E-10 | 72 |
| BP | GO:0009566 | fertilization | 1.89E-08 | 38 |
| BP | GO:0051321 | meiotic cell cycle | 1.94E-08 | 44 |
| BP | GO:0061982 | meiosis I cell cycle process | 6.01E-08 | 29 |
| BP | GO:0007127 | meiosis I | 9.90E-08 | 28 |
| BP | GO:1903046 | meiotic cell cycle process | 1.07E-07 | 36 |
| BP | GO:0140013 | meiotic nuclear division | 5.34E-07 | 33 |
| BP | GO:0007338 | single fertilization | 8.46E-07 | 31 |
| BP | GO:0007018 | microtubule-based movement | 8.46E-07 | 52 |
| BP | GO:0140014 | mitotic nuclear division | 8.46E-07 | 45 |
| BP | GO:0007059 | chromosome segregation | 4.30E-05 | 45 |
| BP | GO:0007140 | male meiotic nuclear division | 5.30E-05 | 14 |
| BP | GO:0060271 | cilium assembly | 5.30E-05 | 49 |
| BP | GO:0000070 | mitotic sister chromatid segregation | 5.30E-05 | 28 |
| BP | GO:0003341 | cilium movement | 5.30E-05 | 28 |
| BP | GO:0007141 | male meiosis I | 5.30E-05 | 10 |
| BP | GO:0001539 | cilium or flagellum-dependent cell motility | 5.30E-05 | 24 |
| BP | GO:0060285 | cilium-dependent cell motility | 5.30E-05 | 24 |
| BP | GO:0044782 | cilium organization | 9.34E-05 | 50 |
| BP | GO:0098813 | nuclear chromosome segregation | 0.000246 | 37 |
| BP | GO:0000819 | sister chromatid segregation | 0.000263 | 30 |
| BP | GO:0035036 | sperm-egg recognition | 0.000264 | 15 |
| BP | GO:0045071 | negative regulation of viral genome replication | 0.000287 | 14 |
| BP | GO:0007339 | binding of sperm to zona pellucida | 0.000869 | 12 |
| BP | GO:0048002 | antigen processing and presentation of peptide antigen | 0.0012 | 28 |
| BP | GO:0030317 | flagellated sperm motility | 0.002095 | 18 |
| BP | GO:0097722 | sperm motility | 0.002095 | 18 |
| BP | GO:0001675 | acrosome assembly | 0.002374 | 7 |
| BP | GO:0070286 | axonemal dynein complex assembly | 0.00293 | 10 |
| BP | GO:0009636 | response to toxic substance | 0.003068 | 31 |
| BP | GO:0002495 | antigen processing and presentation of peptide antigen via MHC class II | 0.003268 | 18 |
| BP | GO:0035082 | axoneme assembly | 0.003434 | 15 |
| BP | GO:0002504 | antigen processing and presentation of peptide or polysaccharide antigen via MHC class II | 0.003545 | 18 |
| BP | GO:0060294 | cilium movement involved in cell motility | 0.003608 | 19 |
| BP | GO:0019882 | antigen processing and presentation | 0.004532 | 30 |
| BP | GO:0001578 | microtubule bundle formation | 0.004875 | 18 |
| BP | GO:0009988 | cell-cell recognition | 0.005691 | 15 |
| BP | GO:0019886 | antigen processing and presentation of exogenous peptide antigen via MHC class II | 0.005779 | 17 |
| BP | GO:0043046 | DNA methylation involved in gamete generation | 0.006728 | 7 |
| BP | GO:0007292 | female gamete generation | 0.007111 | 20 |
| BP | GO:0019884 | antigen processing and presentation of exogenous antigen | 0.007291 | 25 |
| BP | GO:0045069 | regulation of viral genome replication | 0.007928 | 15 |
| BP | GO:0002478 | antigen processing and presentation of exogenous peptide antigen | 0.009107 | 24 |
| BP | GO:0070192 | chromosome organization involved in meiotic cell cycle | 0.009107 | 13 |
| BP | GO:0051783 | regulation of nuclear division | 0.00969 | 20 |
| BP | GO:0007131 | reciprocal meiotic recombination | 0.00969 | 12 |
| BP | GO:0140527 | reciprocal homologous recombination | 0.00969 | 12 |
| BP | GO:0008608 | attachment of spindle microtubules to kinetochore | 0.012147 | 9 |
| BP | GO:0007051 | spindle organization | 0.012691 | 24 |
| BP | GO:0035825 | homologous recombination | 0.012691 | 12 |
| BP | GO:0045143 | homologous chromosome segregation | 0.012691 | 12 |
| BP | GO:0006090 | pyruvate metabolic process | 0.013316 | 21 |
| BP | GO:0051983 | regulation of chromosome segregation | 0.014461 | 15 |
| BP | GO:0007088 | regulation of mitotic nuclear division | 0.014461 | 17 |
| BP | GO:1905818 | regulation of chromosome separation | 0.01617 | 13 |
| BP | GO:0042447 | hormone catabolic process | 0.018594 | 5 |
| BP | GO:0010639 | negative regulation of organelle organization | 0.019096 | 37 |
| BP | GO:0007340 | acrosome reaction | 0.020179 | 9 |
| BP | GO:0070193 | synaptonemal complex organization | 0.024634 | 7 |
| BP | GO:1903900 | regulation of viral life cycle | 0.026815 | 20 |
| BP | GO:0021681 | cerebellar granular layer development | 0.027886 | 5 |
| BP | GO:0042445 | hormone metabolic process | 0.027886 | 26 |
| BP | GO:0098754 | detoxification | 0.027886 | 19 |
| BP | GO:0034587 | piRNA metabolic process | 0.027886 | 6 |
| BP | GO:0007129 | homologous chromosome pairing at meiosis | 0.030493 | 10 |
| BP | GO:0000086 | G2/M transition of mitotic cell cycle | 0.034018 | 29 |
| BP | GO:1902115 | regulation of organelle assembly | 0.036064 | 23 |
| BP | GO:0007289 | spermatid nucleus differentiation | 0.036651 | 6 |
| BP | GO:0048525 | negative regulation of viral process | 0.038752 | 14 |
| BP | GO:0044839 | cell cycle G2/M phase transition | 0.045452 | 30 |
| BP | GO:0150146 | cell junction disassembly | 0.047852 | 6 |
| BP | GO:0098869 | cellular oxidant detoxification | 0.049642 | 15 |
| BP | GO:0006869 | lipid transport | 0.049642 | 44 |
| CC | GO:0031514 | motile cilium | 2.00E-11 | 43 |
| CC | GO:0097729 | 9+2 motile cilium | 3.96E-08 | 28 |
| CC | GO:0001669 | acrosomal vesicle | 4.85E-08 | 27 |
| CC | GO:0036126 | sperm flagellum | 8.24E-08 | 26 |
| CC | GO:0005874 | microtubule | 2.05E-05 | 52 |
| CC | GO:0005814 | centriole | 4.15E-05 | 25 |
| CC | GO:0000922 | spindle pole | 7.39E-05 | 27 |
| CC | GO:0001673 | male germ cell nucleus | 0.000646 | 7 |
| CC | GO:0005930 | axoneme | 0.000806 | 21 |
| CC | GO:0097014 | ciliary plasm | 0.000927 | 21 |
| CC | GO:0030667 | secretory granule membrane | 0.001752 | 36 |
| CC | GO:0002080 | acrosomal membrane | 0.002577 | 8 |
| CC | GO:0099568 | cytoplasmic region | 0.003222 | 31 |
| CC | GO:0005819 | spindle | 0.003222 | 41 |
| CC | GO:0043073 | germ cell nucleus | 0.003222 | 7 |
| CC | GO:0032838 | plasma membrane bounded cell projection cytoplasm | 0.00435 | 27 |
| CC | GO:0005875 | microtubule associated complex | 0.004724 | 22 |
| CC | GO:0097225 | sperm midpiece | 0.005555 | 8 |
| CC | GO:0031091 | platelet alpha granule | 0.005894 | 15 |
| CC | GO:0062023 | collagen-containing extracellular matrix | 0.005894 | 43 |
| CC | GO:0005858 | axonemal dynein complex | 0.006256 | 7 |
| CC | GO:0036064 | ciliary basal body | 0.006345 | 21 |
| CC | GO:0000793 | condensed chromosome | 0.007928 | 26 |
| CC | GO:0042611 | MHC protein complex | 0.009705 | 7 |
| CC | GO:0000794 | condensed nuclear chromosome | 0.011071 | 14 |
| CC | GO:0033391 | chromatoid body | 0.012483 | 5 |
| CC | GO:0030286 | dynein complex | 0.019586 | 10 |
| CC | GO:0000228 | nuclear chromosome | 0.024632 | 27 |
| CC | GO:0097228 | sperm principal piece | 0.024632 | 6 |
| CC | GO:0031093 | platelet alpha granule lumen | 0.027778 | 11 |
| CC | GO:0032391 | photoreceptor connecting cilium | 0.027778 | 8 |
| CC | GO:0042613 | MHC class II protein complex | 0.0289 | 5 |
| CC | GO:0061827 | sperm head | 0.0289 | 5 |
| CC | GO:0030496 | midbody | 0.029082 | 22 |
| CC | GO:0000795 | synaptonemal complex | 0.039418 | 8 |
| CC | GO:0099086 | synaptonemal structure | 0.039418 | 8 |
| CC | GO:0005811 | lipid droplet | 0.048175 | 13 |
| MF | GO:0015631 | tubulin binding | 0.00033 | 48 |
| MF | GO:0008017 | microtubule binding | 0.001021 | 37 |
| MF | GO:0004181 | metallocarboxypeptidase activity | 0.010493 | 9 |
| MF | GO:0004601 | peroxidase activity | 0.013349 | 12 |
| MF | GO:0008235 | metalloexopeptidase activity | 0.015412 | 13 |
| MF | GO:0016887 | ATPase activity | 0.015412 | 50 |
| MF | GO:0016684 | oxidoreductase activity, acting on peroxide as acceptor | 0.016188 | 12 |
| MF | GO:0004180 | carboxypeptidase activity | 0.017622 | 10 |
| MF | GO:0003777 | microtubule motor activity | 0.020203 | 13 |
| MF | GO:0004602 | glutathione peroxidase activity | 0.020203 | 7 |
| MF | GO:0008238 | exopeptidase activity | 0.042144 | 15 |
| MF | GO:0016209 | antioxidant activity | 0.042144 | 14 |
| MF | GO:0003779 | actin binding | 0.042144 | 44 |
| MF | GO:0004364 | glutathione transferase activity | 0.042144 | 7 |
| MF | GO:0008237 | metallopeptidase activity | 0.042144 | 23 |
| MF | GO:0003774 | motor activity | 0.042144 | 18 |

GO: Gene Ontology; DEGs: differently expressed genes; BP: biological process; CC: cellular component; MF: molecular function.
