# Supplementary material for: Constructing a seventeen-gene signature model for non-obstructive azoospermia based on integrated transcriptome analyses and WGCNA
Source: Reprod Biol Endocrinol. 2023 Mar 21;21:30. doi: 10.1186/s12958-023-01079-5 (PMC10029246; doi:10.1186/s12958-023-01079-5)
Supplement: Supplementary file 7 — Additional file 7: Supplementary Table 6. KEGG enrichment analysis of common DEGs by GSVA. [file 12958_2023_1079_MOESM7_ESM.docx]

**Supplementary Table 6**

KEGG enrichment analysis of common DEGs by GSVA.

| KEGG pathway | logFC | adj.P.Val | Change |
| --- | --- | --- | --- |
| KEGG_LYSOSOME | 0.520886 | 1.19E-09 | Up |
| KEGG_PYRUVATE_METABOLISM | 0.546268 | 3.51E-09 | Up |
| KEGG_PANCREATIC_CANCER | 0.597113 | 5.94E-09 | Up |
| KEGG_STEROID_HORMONE_BIOSYNTHESIS | 0.693634 | 3.38E-07 | Up |
| KEGG_INTESTINAL_IMMUNE_NETWORK_FOR_IGA_PRODUCTION | 0.734557 | 3.46E-07 | Up |
| KEGG_PROSTATE_CANCER | 0.53645 | 7.30E-07 | Up |
| KEGG_TYPE_I_DIABETES_MELLITUS | 0.727814 | 8.96E-07 | Up |
| KEGG_AUTOIMMUNE_THYROID_DISEASE | 0.727814 | 8.96E-07 | Up |
| KEGG_ALLOGRAFT_REJECTION | 0.727814 | 8.96E-07 | Up |
| KEGG_GRAFT_VERSUS_HOST_DISEASE | 0.727814 | 8.96E-07 | Up |
| KEGG_FOCAL_ADHESION | 0.656884 | 9.56E-07 | Up |
| KEGG_SYSTEMIC_LUPUS_ERYTHEMATOSUS | 0.699322 | 2.40E-06 | Up |
| KEGG_PARKINSONS_DISEASE | 0.411343 | 3.23E-06 | Up |
| KEGG_VASCULAR_SMOOTH_MUSCLE_CONTRACTION | 0.557884 | 3.31E-06 | Up |
| KEGG_COMPLEMENT_AND_COAGULATION_CASCADES | 0.668361 | 4.42E-06 | Up |
| KEGG_ASTHMA | 0.54816 | 8.23E-06 | Up |
| KEGG_VIRAL_MYOCARDITIS | 0.63244 | 1.42E-05 | Up |
| KEGG_REGULATION_OF_ACTIN_CYTOSKELETON | 0.437167 | 1.51E-05 | Up |
| KEGG_DRUG_METABOLISM_CYTOCHROME_P450 | 0.554676 | 1.89E-05 | Up |
| KEGG_METABOLISM_OF_XENOBIOTICS_BY_CYTOCHROME_P450 | 0.533909 | 1.89E-05 | Up |
| KEGG_ANTIGEN_PROCESSING_AND_PRESENTATION | 0.431174 | 2.21E-05 | Up |
| KEGG_ECM_RECEPTOR_INTERACTION | 0.515674 | 2.36E-05 | Up |
| KEGG_ALZHEIMERS_DISEASE | 0.296811 | 2.37E-05 | Up |
| KEGG_LONG_TERM_DEPRESSION | 0.503266 | 3.22E-05 | Up |
| KEGG_PATHWAYS_IN_CANCER | 0.268829 | 3.34E-05 | Up |
| KEGG_CELL_ADHESION_MOLECULES_CAMS | 0.565008 | 6.35E-05 | Up |
| KEGG_PPAR_SIGNALING_PATHWAY | 0.416999 | 0.000105 | Up |
| KEGG_PRION_DISEASES | 0.573575 | 0.000459 | Up |
| KEGG_SMALL_CELL_LUNG_CANCER | 0.378003 | 0.002814 | Up |
| KEGG_ALDOSTERONE_REGULATED_SODIUM_REABSORPTION | 0.32643 | 0.014614 | Up |
| KEGG_LEISHMANIA_INFECTION | 0.318335 | 0.045407 | Up |
| KEGG_MTOR_SIGNALING_PATHWAY | 0.274765 | 0.045407 | Up |
| KEGG_INSULIN_SIGNALING_PATHWAY | -0.52191 | 8.04E-10 | Down |
| KEGG_AMYOTROPHIC_LATERAL_SCLEROSIS_ALS | -0.70115 | 3.64E-07 | Down |
| KEGG_GLYCEROPHOSPHOLIPID_METABOLISM | -0.39033 | 1.02E-05 | Down |
| KEGG_CHEMOKINE_SIGNALING_PATHWAY | -0.32745 | 0.000232 | Down |
| KEGG_JAK_STAT_SIGNALING_PATHWAY | -0.37419 | 0.000828 | Down |
| KEGG_TIGHT_JUNCTION | -0.37531 | 0.003502 | Down |
| KEGG_T_CELL_RECEPTOR_SIGNALING_PATHWAY | -0.33399 | 0.011974 | Down |
| KEGG_APOPTOSIS | -0.23581 | 0.044643 | Down |

KEGG: Kyoto Encyclopedia of Genes and Genomes; DEGs: differentially expressed genes; GSVA: gene set variation analysis; FC: fold change; Change: Up or Down denotes up or down-regulated KEGG pathway of GSVA result in the NOA group compared with the Control group.
