# Supplementary material for: Constructing a seventeen-gene signature model for non-obstructive azoospermia based on integrated transcriptome analyses and WGCNA
Source: Reprod Biol Endocrinol. 2023 Mar 21;21:30. doi: 10.1186/s12958-023-01079-5 (PMC10029246; doi:10.1186/s12958-023-01079-5)
Supplement: Supplementary file 8 — Additional file 8: Supplementary Table 7. Clinical characteristics of patients with NOA and OA. [file 12958_2023_1079_MOESM8_ESM.docx]

**Supplementary Table 7**

Clinical characteristics of patients with NOA and OA.

| Parameters | NOA1 | NOA2 | NOA2 | OA1 | OA2 | OA3 |
| --- | --- | --- | --- | --- | --- | --- |
| Age (year) | 30 | 43 | 28 | 23 | 39 | 30 |
| BMI (kg m^-2^) | 20.20 | 26.23 | 18.11 | 27.89 | 25.43 | 27.76 |
| Testicular volume (ml) ^†^ | 5 | 8 | 6.5 | 10.5 | 12 | 12 |
| Accompanying phenotype | epididymal cyst (right), varicocele (left) | varicocele (left) | - | - | - | - |
| Karyotype | 46, XY | 46, XY | 46, XY | 46, XY | 46, XY | 46, XY |
| Y Chromosome microdeletion | Neg | Neg | Neg | Neg | Neg | Neg |
| Seminal plasma biochemistry | - | - | - | Decreased levels of neutral alpha-glucosidase and fructose | Decreased level of neutral alpha-glucosidase and increased level of elastase | Decreased level of neutral alpha-glucosidase and increased level of elastase |
| Serum FSH (mIU ml^-1^) | 38.41 | 23.54 | 18.29 | 4.72 | 13.05 | 5.36 |
| Serum LH (mIU ml^-1^) | 10.90 | 9.33 | 4.37 | 3.19 | 4.43 | 3.11 |
| Serum T (ng ml^-1^) | 4.15 | 1.40 | NA | 3.43 | 4.33 | 2.53 |
| Serum E_2_ (pg ml^-1^) | 67.3 | 23.00 | NA | 31 | 23.88 | 40.71 |
| Serum P (ng ml^-1^) | 0.10 | - | NA | 0.22 | 0.26 | 0.87 |
| Serum PRL (ng ml^-1^) | 10.19 | 18.06 | NA | 6.24 | 9.10 | 5.37 |
| Previous treatment | - | Anastrozole (3 months) | - | - | - | - |
| History of parotitis/orchitis | Neg | Neg | Neg | Neg | Neg | Neg |
| History of testicular trauma | Neg | Neg | Neg | Neg | Neg | Neg |
| History of bilateral hernia surgery | Neg | Neg | Neg | Neg | Neg | Neg |
| Exposure to environmental toxins/radiation/ chemotherapeutic drug | Neg | Neg | Neg | Neg | Neg | Neg |
| Exposure to high temperature | Neg | Neg | Neg | Neg | Neg | Neg |
| Exposure to drug | Neg | Neg | Neg | Neg | Neg | Neg |
| Smoking | Neg | Neg | Neg | Neg | Neg | Neg |
| Alcohol consumption | Neg | Neg | Neg | Neg | Neg | Neg |
| Consanguine marriage | Neg | Neg | Neg | Neg | Neg | Neg |
| Testicular biopsy | No spermatogenic cells and sperm were seen. | No spermatogenic cells and sperm were seen. | No spermatogenic cells and sperm were seen. | Spermatogenic cells, spermatids and sperms were seen | Spermatogenic cells, spermatids and sperms were seen | Spermatogenic cells, spermatids and sperms were seen |

^†^Testicular volume refers to the mean value of bilateral testicular volume.

NOA: non-obstructive azoospermia; OA: obstructive azoospermia; BMI: body mass index; FSH: follicle‑stimulating hormone (normal range: 1.27-19.26 mIU ml^-1^); LH: luteinizing hormone (normal range: 1.24-8.62 mIU ml^-1^); T: testosterone (normal range: 1.75-7.81 ng ml^-1^); E_2_: estradiol (normal range: ≤ 38.95 pg ml^-1^); P: progesterone (normal range: 0.14-2.06 ng ml^-1^); PRL: prolactin (normal range: 2.64-13.13 ng ml^-1^); Neg: negative; NA: not available.
